# Mutation of *wbtJ*, a *N*-formyltransferase involved in O-antigen synthesis, results in biofilm formation, phase variation and attenuation in *Francisella tularensis*

**DOI:** 10.1099/mic.0.001437

**Published:** 2024-02-29

**Authors:** Kevin D. Mlynek, Ronald G. Toothman, Elsie E. Martinez, Ju Qiu, Joshua B. Richardson, Joel A. Bozue

**Affiliations:** ^1^​ Bacteriology Division, US ARMY Medical Research Institute of Infectious Diseases (USAMRIID), Frederick, MD, USA; ^2^​ Regulated Research Administration Division, USAMRIID, Frederick, MD, USA; ^3^​ Center for Genome Sciences, USAMRIID, Frederick, MD, USA

**Keywords:** biofilm, *Francisella tularensis*, LPS, O-antigen, phase variation, SNP reversion, tularaemia, virulence, WbtJ

## Abstract

Two clinically important subspecies, *Francisella tularensis* subsp. *tularensis* (type A) and *F. tularensis* subsp. *holarctica* (type B) are responsible for most tularaemia cases, but these isolates typically form a weak biofilm under *in vitro* conditions. Phase variation of the *F. tularensis* lipopolysaccharide (LPS) has been reported in these subspecies, but the role of variation is unclear as LPS is crucial for virulence. We previously demonstrated that a subpopulation of LPS variants can constitutively form a robust biofilm *in vitro,* but it is unclear whether virulence was affected. In this study, we show that biofilm-forming variants of both fully virulent *F. tularensis* subspecies were highly attenuated in the murine tularaemia model by multiple challenge routes. Genomic sequencing was performed on these strains, which revealed that all biofilm-forming variants contained a lesion within the *wbtJ* gene, a formyltransferase involved in O-antigen synthesis. A Δ*wbtJ* deletion mutant recapitulated the biofilm, O-antigen and virulence phenotypes observed in natural variants and could be rescued through complementation with a functional *wbtJ* gene. Since the spontaneously derived biofilm-forming isolates in this study were a subpopulation of natural variants, reversion events to the *wbtJ* gene were detected that eliminated the phenotypes associated with biofilm variants and restored virulence. These results demonstrate a role for WbtJ in biofilm formation, LPS variation and virulence of *F. tularensis*.

## Introduction

*Francisella tularensis* is an intracellular pathogen that is endemic throughout much of the northern hemisphere and is the causative agent of tularaemia [1, 2]. While only a few hundred cases occur per year in humans in the USA [3], tularaemia can be life-threatening if left untreated [4]. Two *Francisella* subspecies are almost exclusively responsible for causing illness in humans: *F. tularensis* subsp. *tularensis* (type A) and *F. tularensis* subsp. *holarctica* (type B). In North America, type A isolates tend to be more virulent and are most often transmitted by deer flies and tick vectors in arid environments [1, 2, 5, 6]. On the other hand, type B isolates are usually less virulent, are distributed throughout much of Eurasia, and are more often associated with aquatic environments and transmitted through mosquitoes [7–10]. From a biodefence perspective, *F. tularensis* poses a significant threat as a bioweapon due to its notoriously low infectious dose, aerosizable nature and high morbidity [5]. While a live vaccine strain (LVS) has previously been developed, approval by the US Food and Drug Administration (FDA) for use in the USA has not occurred due to multiple concerns, such as an unknown ancestral history, instability of colony morphology and potential reversion to a virulent form [11, 12].

**Table 1.** Bacterial strains used in this study

Strain	Strain characteristics	Source
*Escherichia coli* NEB5-α	Used for routine plasmid construction in this study	New England Biolabs
FRAN244	Schu S4 (type A1)	BEI (NR-10492) [20]
244 BF-1	Grey variant; biofilm-forming isolate of FRAN244	[28]
FRAN255	Isolate from human pleura in 2015 (type B2)	BRMR* [40]
255 BF-1	Grey variant; biofilm-forming isolate of FRAN255	[28]
255 BF-4	Grey variant; biofilm-forming isolate of FRAN255	[28]
LVS	Live vaccine strain	USAMRIID Repository
LVS isolate #11	Grey variant; non-biofilm-forming isolate of LVS	[28]
LVS isolate #13	Grey variant; non-biofilm-forming isolate of LVS	[28]
LVS isolate #14	Grey variant; non-biofilm-forming isolate of LVS	[28]
LVS isolate #15	Grey variant; biofilm-forming isolate of LVS	[28]
LVS isolate #22	Grey variant; biofilm-forming isolate of LVS	[28]
LVS isolate #26	Grey variant; biofilm-forming isolate of LVS	[28]
LVS isolate #27	Grey variant; biofilm-forming isolate of LVS	[28]
LVS isolate #38	Grey variant; biofilm-forming isolate of LVS	[28]
LVS Δ*wbtJ*	In-frame deletion of FTL_0602 in LVS	This study
LVS Δ*wbtJ*/pMP814	In-frame deletion of FTL_0602 in LVS with pMP814 (KanR)	This study
LVS Δ*wbtJ*/pKM43	In-frame deletion of FTL_0602 in LVS with pKM43 (KanR)	This study
LVS isolate #15/pMP814	LVS isolate #15 with pMP814 (KanR)	This study
LVS isolate #15/pKM43	LVS isolate #15 with pKM43 (KanR)	This study
#15-R1	Reverted colony from LVS isolate #15; blue variant, non-biofilm-forming	This study
#15-R2	Reverted colony from LVS isolate #15; blue variant, non-biofilm-forming	This study
#15-R3	Reverted colony from LVS isolate #15; blue variant, non-biofilm-forming	This study
Isolate #15 - MP-1 to 30	Strains isolated from mice challenged with LVS isolate #15	This study
LVS Δ*wbtJ* – MP-1 to 5	Strains isolated from mice challenged with LVS Δ*wbtJ*	This study

*Biodefense Reference Material Repository.

**Table 2.** LD_50_ and TTD for mice challenged intranasally with type A or type B biofilm-forming variants

		**244 BF-1**	**255 BF-1**	**255 BF-4**
	LD_50_ (c.f.u.)	317	> 460 000	2152
Median TTD (Days)	1–10	>14	>14	>14
	10–100	>14	>14	>14
	100–1000	>14	>14	>14
	1000–10 000	8	>14	11
	10,000–100,000	7	>14	9
	>100 000	7	>14	8
				
Pair-Wise Comparisons (Wald-test)*	vs. FRAN244	*P*=0.0036		
	vs. FRAN255		*P*<0.0001	*P*<0.0001

*Challenge dose for the parental strains was 105 c.f.u. (FRAN244) and 8 c.f.u. (FRAN255).

P values for comparisons among strains were determined by Wald Chi-square tests based on a Cox regression model.

ns, not significant (P>0.05); TTD, time to death.

**Table 3.** SNP analysis of biofilm-forming variants to identify mutated genes

Strain	Position	TYPE	REF	ALT	Strand	NT pos	AA pos	Effect	Mutation	Gene	Product
244 BF-1	1 503 971	snp	G	A	–	434/726	145/241	missense	Ser145Phe	*wbtJ*	dTDP-4-amino-4,6-dideoxyglucose formyltransferase
											
255 BF-1	551 888	snp	A	T	+	1368/1686	456/561	missense	Leu456Phe	*yheS*	putative ABC transporter ATP-binding protein YheS
	1 016 152	snp	A	T	–	13/258	5/85	missense	Leu5Ile		hypothetical protein
	1 099 427	snp	T	A	+	72/141	24/46	synonymous	Pro24Pro		hypothetical protein
	1 429 136	insert	T	TA	+	433/447	145/148	frameshift	Tyr145fs		IS630 family transposase ISFtu1
	1 503 160	delete	TC	T	–	370/726	124/241	frameshift	Glu124fs	*wbtJ*	dTDP-4-amino-4,6-dideoxyglucose formyltransferase
	1 608 120	delete	AT	A							intergenic
											
255 BF-4	460 860	snp	C	G	+	383/1347	128/448	nonsense	Ser128*	*dtpA1*	Dipeptide and tripeptide permease A
	1 249 956	snp	T	G	–	441/465	147/154	synonymous	Ile147Ile	*dtpD*	Dipeptide permease D
	1 503 065	insert	C	CT	–	465/726	155/241	frameshift	Glu156fs	*wbtJ*	dTDP-4-amino-4,6-dideoxyglucose formyltransferase
	1 643 954	snp	T	C				unknown			intergenic
	1 736 592	snp	A	C				unknown			intergenic

Tularaemia is known to manifest clinically in several forms in humans (ulceroglandular, glandular, oculoglandular, oropharyngeal, pneumonic, typhoidal) that can often be indicative of the route of exposure [8]. The most common forms of tularaemia are ulceroglandular and glandular, which are characterized by an infection at the bacterial entry site and are often attributed to bites from insects harbouring *F. tularensis* or the handling of an infected animal [2, 13]. Pneumonic tularaemia, occurring from aerosol exposure or hematogenous spread, is the most severe form of the disease and is associated with higher mortality rates [4]. As an intracellular pathogen, *F. tularensis* is able to avoid immune detection to invade a variety of host cells, most often macrophages and hepatocytes [14, 15]. It is generally agreed that the unique structure of *F. tularensis* lipopolysaccharide (LPS) allows the bacteria to avoid the canonical TLR4 signalling pathway that alerts immune cells to bacterial invasion [16, 17]. Further, antigenic variation of the LPS structure, namely lipid A and the O antigen (O-Ag), has been shown to modulate host factors within macrophages, highlighting the importance LPS in the pathogenesis of *F. tularensis* [18, 19].

Early characterization studies on *F. tularensis* noted that surface factors were able to vary, which affected the colony morphology, virulence and immunogenic response [20–22]. This phenomenon was originally described as blue/grey phase variation on the basis of colony appearance under oblique lighting [21]. Blue variants were found to be the virulent form of *F. tularensis*, while some level of attenuation was observed in grey variants. Importantly, the reversion of grey variants to a blue form has also been reported, suggesting that variants cannot be assumed to be locked in an attenuated state [18, 21]. This phenotypic switching between blue and grey forms is readily observed in both virulent type A and B strains, as well as LVS [20, 23]. To date, it is unknown whether this variation only happens in a laboratory setting or if it is relevant to the environmental survival of *F. tularensis*. However, understanding this variation in *F. tularensis* is important because in the past, vaccine lots of LVS have displayed reduced immunogenicity in humans due to an inexplicable rise in grey variants during batch production [20, 24]. While the cellular factors that govern blue/grey variation in *F. tularensis* are still largely unknown, this phenotype has been attributed to alterations to the LPS molecule and/or the O-Ag that can be also incorporated into the capsule [18, 23, 25]. Culture conditions, such as growth medium, pH, inoculum density and culture duration, have been shown to influence the frequency of blue to grey variation, which has led to the working hypothesis that this variation can enable environmental survivorship for this intracellular pathogen [21]. Further supporting this hypothesis, the relative amount of O-Ag displayed on the cell surface has been reported to be influenced by growth environment [26].

In recent years, studies have convincingly shown that fully virulent type A and B isolates can produce a robust biofilm, and there is likely a link between biofilm and environmental survivorship for these isolates [27–29]. However, the ability of *F. tularensis* to form biofilm in a natural setting remains unknown. We previously demonstrated that *in vitro* biofilm formation by *F. tularensis* was dependent on a subpopulation of grey variants that constitutively produce a robust biofilm [28]. In most pathogens, biofilm formation is considered to be a virulence factor because adhesion to host tissues is often a prerequisite for seeding an infection. Biofilm also contributes to an active infection as the extracellular matrix provides protection from the host immune system by masking bacterial surface features and pathogen-associated molecular patterns (PAMPs) and armouring the bacteria against antimicrobial peptides (AMPs) as well as antibiotics [30]. Perhaps the most defined role of biofilm in pathogenesis is the perpetuation of an infection, leading to chronic disease or recalcitrance [31, 32]. However, it is unclear what role, if any, biofilm formation may play in pathogenesis for *F. tularensis*, especially considering that phase variation typically results in attenuation.

In this study, we take advantage of spontaneously laboratory-derived grey variants that constitutively form biofilm in an effort to determine the effect these events may have on pathogenesis in *F. tularensis*. We demonstrate that constitutive biofilm-forming variants for both type A and B isolates of *F. tularensis* are highly attenuated *in vivo* using a murine tularaemia model and multiple challenge routes. While the full genetic mechanism responsible for blue/grey variation remains elusive, we determined that the variants utilized in this study were the result of spontaneous mutations within LPS biosynthesis genes and specifically identified the mutation of the *wbtJ* gene as responsible for the robust biofilm production. To demonstrate the link between WbtJ with blue/grey variation and biofilm formation, an allelic exchange deletion mutant in this gene was constructed and recapitulated the robust biofilm phenotype. In addition, this mutant was shown to be completely attenuated following pneumonic challenge, suggesting that WbtJ is essential for virulence. Lastly, we provide evidence that reversion of a natural grey variant to a blue form can occur *in vivo*, allowing *F. tularensis* to revert from an avirulent to a virulent form within a host.

## Methods

### Bacterial strains and culture conditions

The bacterial strains used in this study are listed in Table 1. Throughout this paper, the terms ‘type A’ or ‘type B’ are used to refer to the respective subspecies, while ‘*F. tularensis’* is used to refer to both subspecies (subsp. *tularensis* and subsp. *holarctica*). *F. tularensis* species were routinely cultured on enriched chocolate agar plates (Remel) at 37 °C. For liquid culture, either brain heart infusion (BHI) broth supplemented with 1 % IsoVitaleX (Becton Dickinson) or Chamberlain’s defined medium (CDM) at pH 6.2 [33] were used for growth.

All molecular cloning and plasmid construction were carried out using NEB5-α electrocompetent *E. coli* (New England Biolabs). When necessary, kanamycin was added to the culture medium at 50 µg ml^−1^ for *E. coli* or 10 µg ml^−1^ for *F. tularensis* to maintain selection in plasmid-containing strains. In instances where reversion or variant isolates were detected, bacteria were streak purified on chocolate agar and confirmed to be *F. tularensis* by PCR of *wbtJ* (Ft-specific gene; oKM189 and oKM192) prior to further experimentation.

### Genome sequencing and SNP detection

Genomic DNA was isolated using a MasterPure isolation kit (Epicentre) following the manufacturer’s protocols. Sequencing libraries were prepared using the Illumina DNA prep kit and sequenced on the Illumina NextSeq 2000 platform using a P3 300 cycle kit. Sequencing libraries for the Oxford Nanopore Mk1C MinION were prepared using the LSK109 ligation sequencing kit and sequenced on version 9 flow cells. Raw reads were quality-trimmed using Trimmomatic v0.38 [34]. Both Illumina and Nanopore reads were used to generate *de novo* assemblies via Unicycler [35]. Mauve was used for whole-genome alignments [36]. Single-nucleotide polymorphism (SNP) analysis and strain consensus generation were performed using snippy v4.6.0 [37] and default settings. The reference strain used was either the *Francisella holarctica* LVS reference (GenBank: CP009694.1), FRAN244 (CP073128.1) or FRAN255 (CP073125.1), depending on the background of the parental isolate. Sequence data from the biofilm-forming variant genomes were uploaded under BioProject number PRJNA970844.

### Construction of a Δ*wbtJ* mutant and complementation

Primers and plasmids used in this study are listed in Tables S1 and S2, available in the online version of this article. Flanking regions of *wbtJ* (FTL_0602) were amplified using primers oKM189 and oRGT_wbtJ_SOE1R to create a 520 upstream fragment and primers oRGT_wbtJ_SOE2F and oKM192 to create a 524 downstream fragment. Overlapping PCR was used to splice the two amplicons together, resulting in a 1031 bp fragment lacking the coding sequence of *wbtJ*. BamHI and EcoRV restriction sites engineered into the primers were used to ligate the fragment into the allelic exchange vector pEDL50 [38]. The resulting plasmid (pKM41) was confirmed by sequencing and electroporated into electrocompetent *Francisella*. Briefly, bacteria were grown to mid-log phase in BHI, washed several times with ice-cold 0.5M sucrose and aliquoted into 50 µl samples. Approximately 1 µg of purified plasmid (pKM41) was electroporated using a Bio-Rad Gene Pulser Xcell set to 2.5kV, 25 µF and 600 Ω. Samples were outgrown in BHI containing IsoVitalex for up to 4 h at 37 °C and plated onto chocolate agar with 10 µg ml^−1^ kanamycin. Colonies required 4–5 days to appear and were confirmed as co-integrates by PCR. The integrated plasmid was cured from co-integrates by overnight passage in BHI followed by serial dilution and plating on chocolate agar with 5 % sucrose. Purified colonies were screened for the Δ*wbtJ* allele using PCR and sequencing.

For complementing the *wbtJ* deletion mutant and LVS isolate #15 (a spontaneous grey variant and biofilm former), the 726 bp coding sequence of *wbtJ* was amplified using primers oKM192 and oKM193 that contained engineered BamHI and EcoRV restriction sites. The resulting fragment was digested and ligated into pMP814 [39], which contains a *blaB* promoter to drive the expression of *wbtJ* for trans-complementation studies. The resulting plasmid (pKM43) was electroporated into the listed *F. tularensis* strains and selected on chocolate agar plates containing kanamycin. Transformants were confirmed by plasmid extraction followed by PCR to confirm the presence of the intact *wbtJ* gene.

### Static biofilm assay

Biofilm formation was assessed by crystal violet staining as previously described [28]. Briefly, *F. tularensis* was cultured for 24 h on chocolate agar and resuspended to an OD_600_ of ~0.3 in phosphate-buffered saline (PBS). Bacterial suspensions were diluted 1 to 10 into CDM (~10^8^ c.f.u.), seeded into CoStar polystyrene 96-well plates and incubated at 37 °C for the indicated length of time. Sterility wells were included in each experiment, and peripheral wells were avoided to minimize edge effect. Prior to biofilm staining, the OD_600_ was measured, after which plates were aspirated, washed 3× with PBS to remove planktonic cells and fixed with 100 % ethanol for 30 min at room temperature. Following ethanol fixation, 0.1 % crystal violet (w/v) was added to each well for 15 min and washed 3× with PBS, after which the remaining crystal violet stain was solubilized in 33 % acetic acid. The OD_600_ was measured to quantify crystal violet staining as an indicator of biofilm formation. When necessary, samples were diluted 1 : 10 in 33 % acetic acid to ensure that OD readings were within the linear range. At least four technical replicates were averaged in each experiment. All data reported are the result of at least three individual experiments. Throughout this paper, strains that formed a robust uniform biofilm within 24–48 h are referred to as ‘biofilm formers’, while strains referred to as ‘non-biofilm formers’ did not form a biofilm within this time frame.

### Macrophage assays

In assays involving macrophages, J774A.1 cells (American Type Culture Collection) were cultured at 37 °C under 5 % CO_2_ in Dulbecco’s modified Eagle’s medium containing glucose, l-glutamine and sodium pyruvate at 4.5 g/L and 10 % foetal bovine serum. To achieve a confluent monolayer, plates were seeded 1 day prior to experiments in 24-well plates at a density of 5×10^5^ cells per well. Immediately prior to infection, *F. tularensis* was resuspended from 18 h plates to an OD_600_ of ~0.3 in PBS and diluted 1 : 5 into tissue culture medium to serve as a bacterial inoculum. No difference was detected for c.f.u. among strains when plating from a normalized OD_600_. To infect macrophages, tissue culture medium was aspirated from the J774A.1 cells and replaced with 200 µl of bacterial inoculum to achieve an m.o.i. of ~100 : 1, which was confirmed by c.f.u. enumeration of the inoculum. At 2 h postinfection, plates were washed 3× with PBS, after which tissue culture medium containing gentamicin (25 µg ml^−1^) was added. To obtain c.f.u. recovery at the indicated time, the infected monolayer of J774A.1 cells was washed 3× with PBS and lysed using 200 µl of sterile water and vigorous scraping. The recovered bacterial sample was diluted 1 : 5 into PBS and serial diluted to allow enumeration on chocolate agar plates. In each experiment, three replicate wells were plated in duplicate to obtain the c.f.u. recovery for an experiment. These data shown are the results of at least three independent experiments.

### Detection of LPS

Whole-cell extracts were prepared from LVS grown for 18 h on chocolate agar plates by resuspending bacteria to an OD_600_ of ~0.5 in PBS. A 1 ml aliquot of each sample was pelleted, resuspended in 1× NuPage LDS gel loading buffer, and boiled for 15 min. c.f.u. were determined for each sample to ensure that equal amounts of bacteria were present in each sample prior to resuspension in gel loading buffer. For western analysis, samples were fractionated on a NuPage Novex 4–12 % Bis–Tris gels, transferred onto a nitrocellulouse membrane via an iBlot Gel Transfer Device and blocked overnight in 1 % skim milk in Tris-buffered saline+0.05 % Tween-20 (TBST). Samples were probed with mouse monoclonal anti-LPS antibody (FB11; MA1-7388; Invitrogen) and rabbit polyclonal anti-GroEL (Enzo Life Sciences) at 1 : 500 and 1 : 2000 dilutions, respectively. The membrane was washed 4× with TBST and the corresponding secondary antibody was applied at 1 : 10 000. After four additional TBST washes, the blot was visualized using Clarity Western ECL Substrate following the manufacturer’s protocols (Bio-Rad).

### LD_50_ determination

*In vivo* testing of *F. tularensis* strains was performed using BALB/c mice (7–9 weeks old from Charles River Laboratories) as previously described [40]. Briefly, *F. tularensis* grown from an 18 h chocolate agar plate was diluted to a known concentration in PBS. To mimic vector inoculation, mice were challenged with the indicated c.f.u. using 100 µl subcutaneous inoculation (FRAN244 strains) or 50 µl intradermal (FRAN255 strains). To model pneumonic tularaemia, mice were lightly anaesthetized with ketamine, acepromazine and xylazine and inoculated intranasally with a 50 µl challenge dose. Mice were monitored for clinical symptoms several times daily and followed for 14 (FRAN244 and FRAN255 strains) or 21 days post-challenge (LVS strains) and mortality rates were recorded. Humane endpoints were used in accordance with a predetermined endpoint score sheet approved by IACUC. At the end of study, spleens and lungs were harvested and checked for c.f.u. recovery, with the exception of LVS-infected mice, as only spleens were examined.

### Statistical analyses

**Fig. 1.**
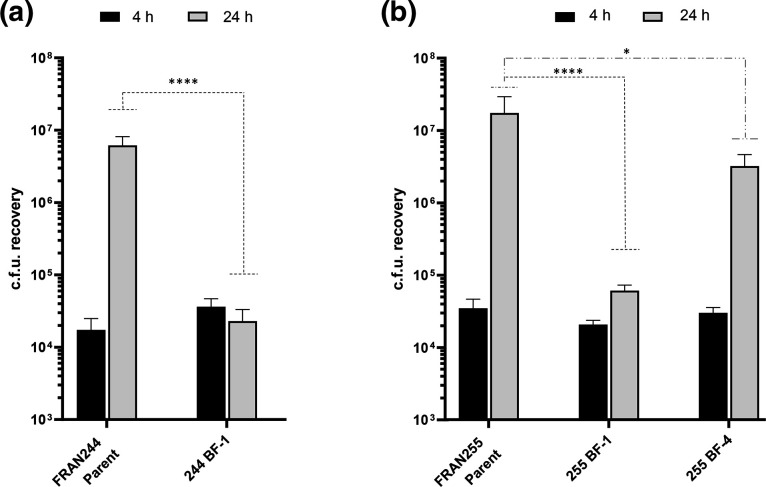
Biofilm variants are altered in their ability to replicate within J774A.1 macrophage-like cells. Gentamicin protection assays were performed using natural biofilm forming variants of (a) FRAN244 (Type A) and (b) FRAN255 (Type B). CFUs were determined at 4 h (black bars) and 24 h (grey bars) post infection. Error bars represent the standard error of the mean from three independent experiments. Significance was assessed using linear mixed effects model on log10 transformed data. *p <0.05, ****p<0.0001.

All experiments described in this paper were performed independently at least three times. Macrophage and biofilm assays (Figs 1, 5–7) were analysed by linear mixed effects model. Analysis was implemented in the GLIMMIX procedure of SAS version 9.4 (SAS Institute, Inc., Raleigh, NC, USA). c.f.u. data were log 10-transformed prior to analysis (Figs 1 and 6). No multiplicity adjustment was applied. LD_50_ values were calculated using a probit model. The median time to death (TTD) was estimated using the Kaplan–Meier method and comparisons between strains were obtained from a log-normal accelerated failure time (AFT) model in the form log(TTD) = *m*×log(dose)+*b*, where *m* and *b* are strain-specific slope and intercept terms (Tables 2 and 5). A Wald chi-square test based on a Cox regression model was used to compare estimated median TTDs at the middle of the dose curve. Analysis was completed in PROC LIFEREG and PROC PROBIT, SAS version 9.4 (SAS Institute Inc, Raleigh, NC, USA).

**Table 4.** Additional SNPs in *wbtJ* identified in independently isolated biofilm forming variants

Strain	Position	TYPE	REF	ALT	NT pos	AA pos	Effect	Mutation
LVS Isolate #15	1 315 079	snp	C	T	459/726	153/241	nonsense	Gln153*
LVS Isolate #22	1 314 645	snp	C	A	23/726	8/241	missense	Thr8Lys
LVS Isolate #26	1 314 764	deletion	AGTGAGATTAAGCCAATAG	A	143/726	48/241	frameshift	Ser48fs
LVS Isolate #27	1 315 196	deletion	ATGTG	A	577/726	193/241	frameshift	Cys193fs
LVS Isolate #31	1 314 969	snp	G	A	347/726	116/241	missense	Gly116Glu
LVS Isolate #38	1 314 834	snp	C	T	212/726	71/241	missense	Ser71Phe

### Data availability

All data underlying the findings and conclusions reported in this paper are fully available within the text or the accompanying Supplementary Material. Genome sequencing is available on GenBank at the time of publication. Additional data are available upon request.

## Results

### Constitutive biofilm variants are impaired for intracellular replication in macrophages

We previously showed that *F. tularensis* can form a robust biofilm within 24 h *in vitro* when blue/grey variation of the O-Ag occurred, suggesting a link between these two poorly understood events [28]. It was also previously demonstrated that grey variants of LVS with alterations to the O-Ag had a survival defect in mouse, rat and human macrophages [23, 25]. Given the importance of intracellular survival for *F. tularensis* pathogenesis, we sought to first assess the ability of biofilm-forming strains (as defined by forming a robust biofilm within 24–48 h) to replicate within host cells. To test this, J774A.1 macrophages were infected with the fully virulent parental wild-type strain and a corresponding spontaneous biofilm-forming variant for a type A lineage (FRAN244/Schu S4 or 244 BF-1) and a type B lineage (FRAN255, 255 BF-1 or 255 BF-4) and assayed c.f.u. recovery at 4 and 24 h post-infection. Two biofilm variants were assayed for FRAN255 as two different O-Ag profiles were previously observed [28]. At 4 h postinfection, similar levels of bacteria were recovered for both the wild-type and the biofilm-forming variants in both type A and B isolate backgrounds (Fig. 1; black bars). However, at 24 h, a significant defect in c.f.u. recovery was detected when comparing the biofilm variants to the parent isolates (Fig. 1, grey bars). More specifically, at 24 h both 244 BF-1 and 255 BF-1 displayed little to no increase compared to the 4 h time point and were significantly different when compared to the increase of the respective parent strains (*P*<0.0001, linear mixed effects model). Interestingly, 255 BF-4 increased approximately 2 logs when compared to the 4 h time point but was still significantly different from the FRAN255 parent for recovery at 24 h (Fig. 1b; *P*<0.05, linear mixed effects model). When these strains were grown extracellularly in broth media, the variants grew as well as the respective parent strain. Taken together, these data suggest that natural biofilm-forming variants are able to infect host cells but differ in their ability to replicate intracellularly.

**Fig. 2.**
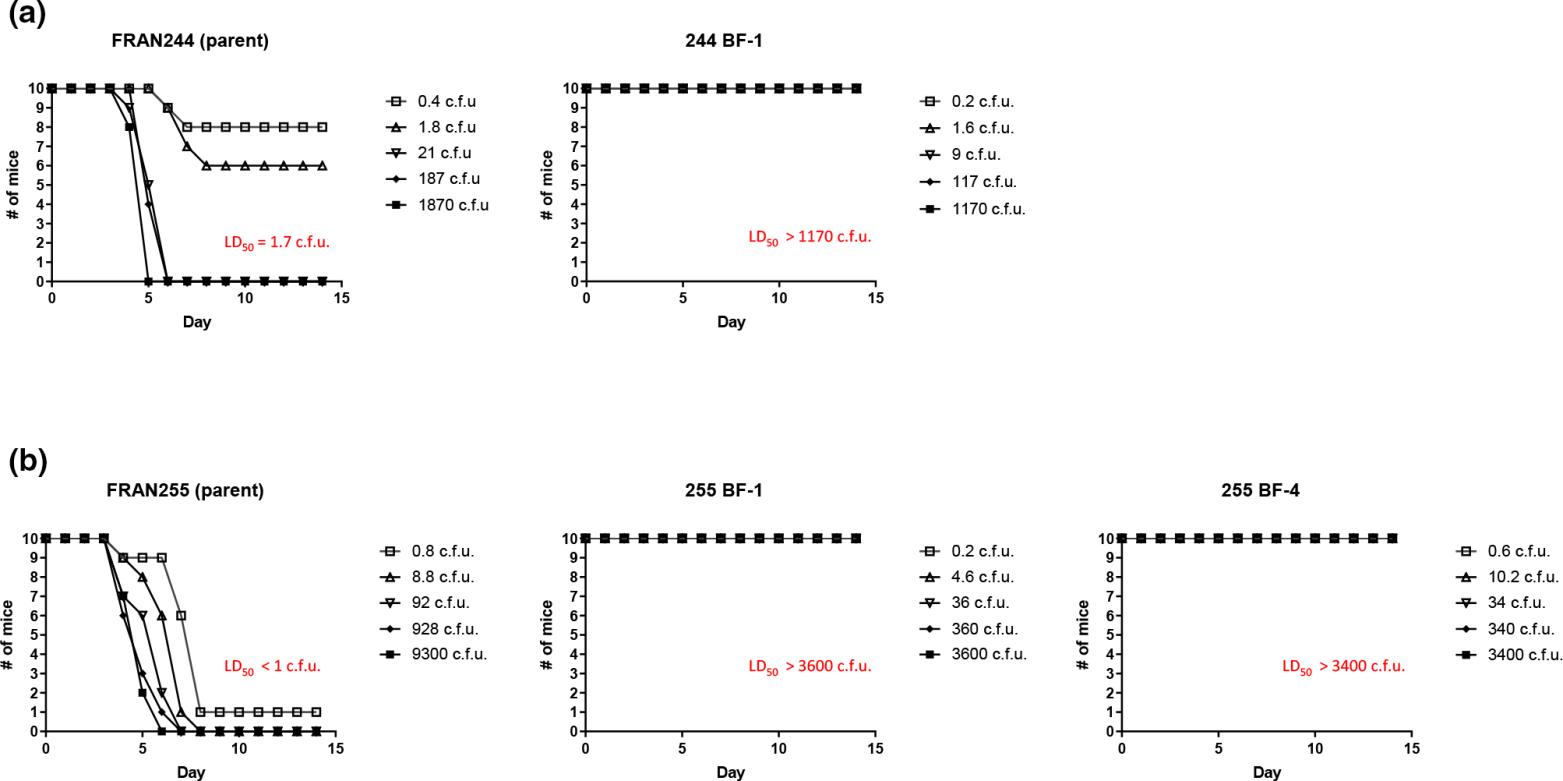
Biofilm forming variants are highly attenuated by challenge mimicking vector exposure in BALB/c mice. Groups of mice (n=10) were challenged with the indicated strain either (a) subcutaneously (FRAN244) or (b) intradermally (FRAN255) and monitored for survivalfor 14 days post challenge. The LD_50_ values for the parent strains were determined as indicated in red. However, all mice challenged with the biofilm forming variants survived at all challenge doses; therefore, an LD_50_ was not able to be determined.

**Fig. 3.**
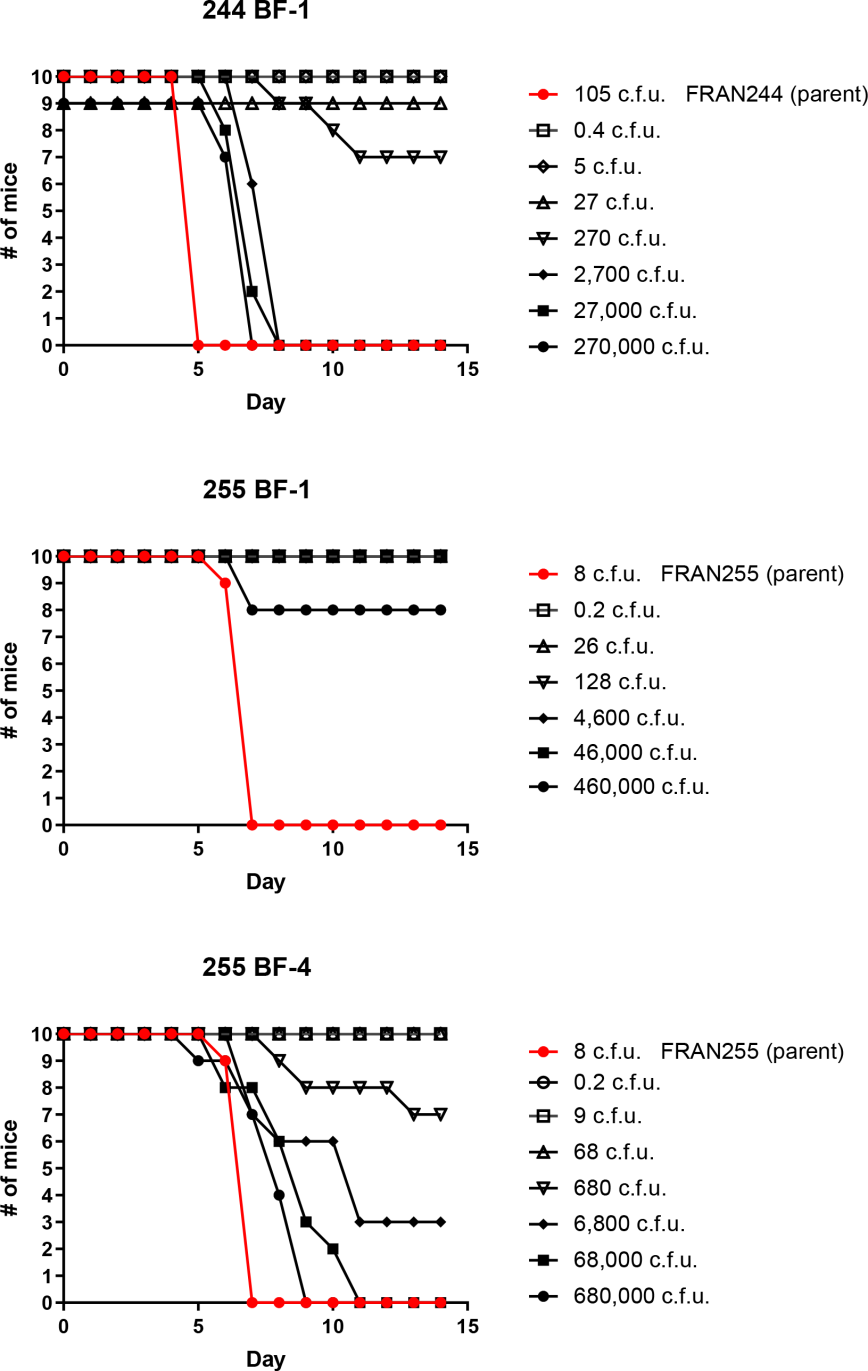
Biofilm forming variants display attenuation in a pneumonic tularemia mouse model. Groups consisting of 10 mice were challenged intranasally with doses ranging from <1 CFU up to 10^6^ CFU for natural biofilm forming variants. As a control, one group of mice was challenged for each the parental strain (red line). Displayed are the survival curves following the mice for 14 days post challenge. The accompanying LD_50_ values and the median time-to-death calculated from these data and are displayed in Table 2.

**Fig. 4.**
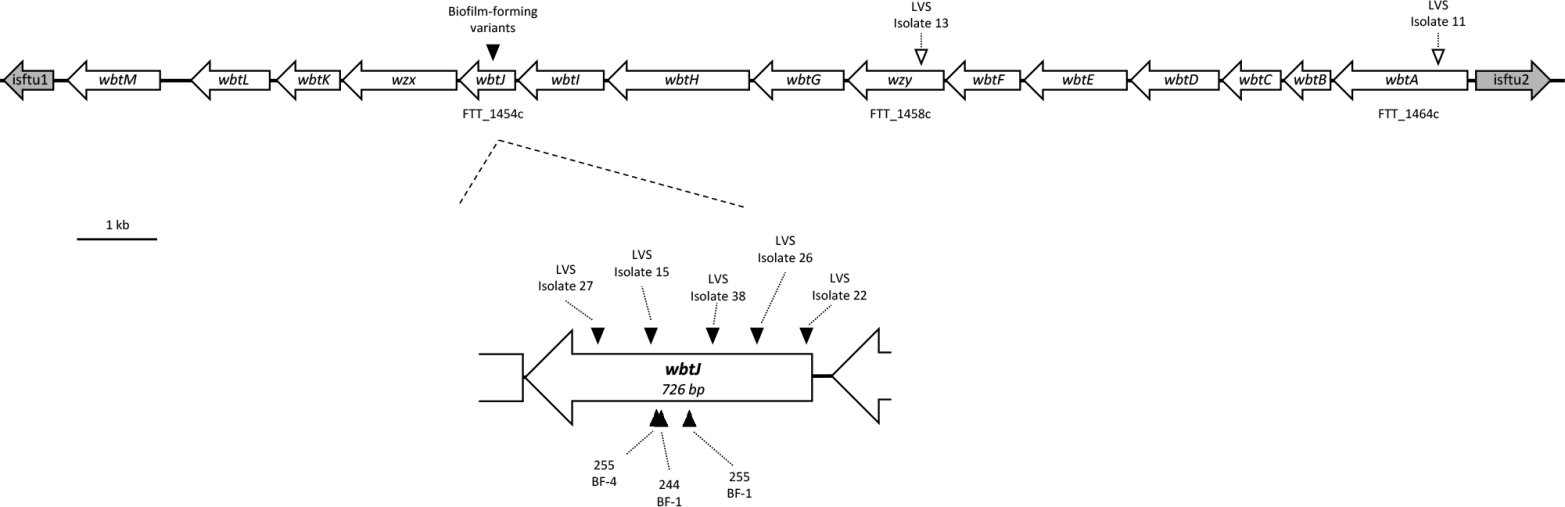
Organization of the *wbt* O-antigen biosynthesis gene cluster highlighting mutations identified in grey variants used in this study. The FRAN244 (Schu S4) genome (CP0731238.1) was used as a reference to create a gene map showing organization of the *wbt* gene cluster. Diagram is drawn to scale; bar indicates 1 kb. Schu S4 locus tags are provided for genes in which mutations were identified. The *wbtJ* gene was expanded to show the relative position of each mutation identified in the gene. Triangles indicate lesions identified in isolates sequenced in this study with the color indicating biofilm status; blackfill indicates biofilm forming while white fill indicates a non-biofilm forming isolate. Strain names are provided for reference.

**Fig. 5.**
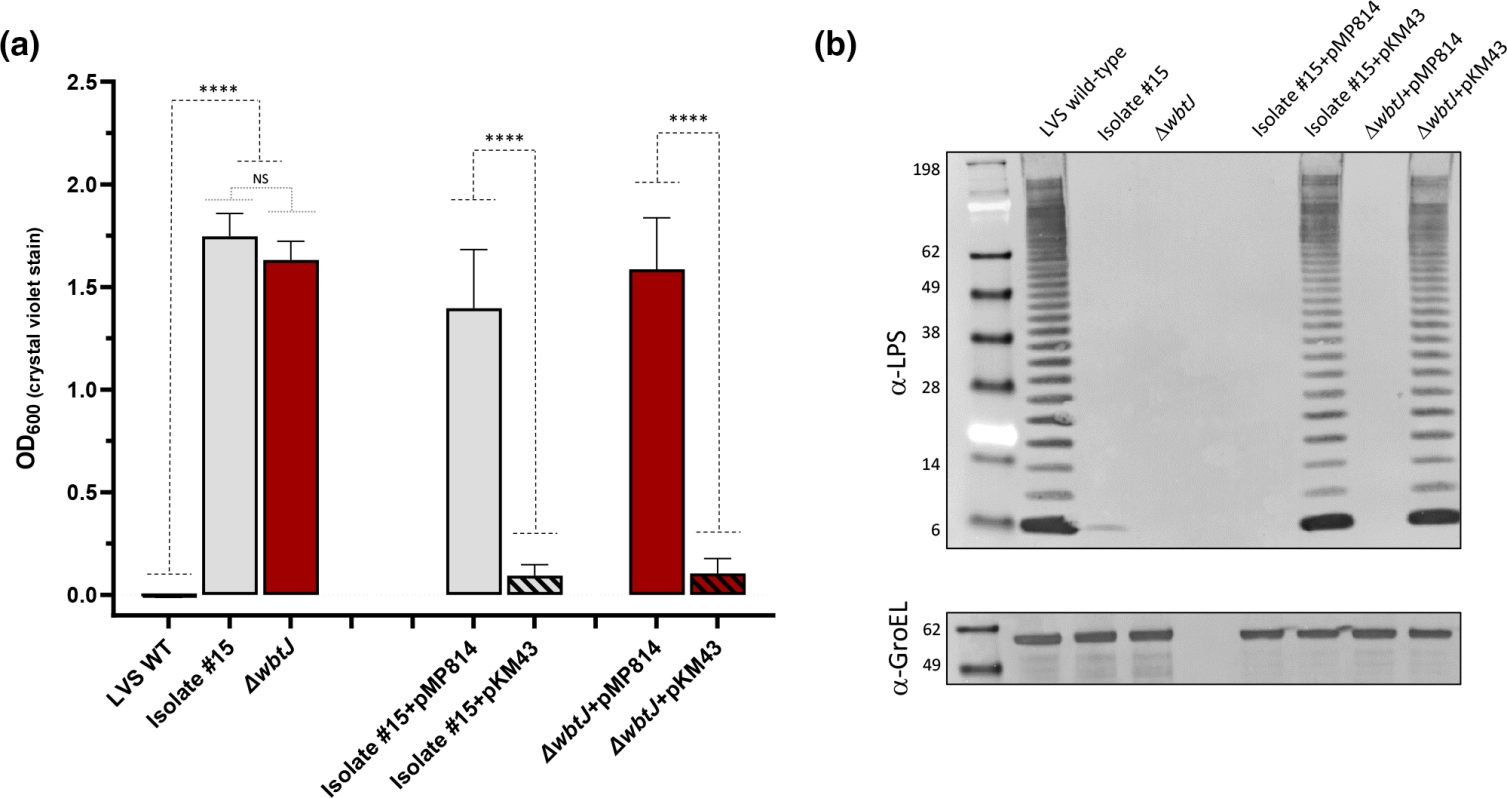
An in-frame deletion of *wbtJ* recapitulates the biofilm and O-Ag phenotypes observed in natural variants and is rescued by complementation. (a) Biofilm formation of the LVS wild-type (black bar), Isolate 15 (grey bars) and a Δ*wbtJ* (red bars) was assayed by crystal violet staining after 1 day of growth in CDM. Biofilm was also assayed in these strains for complementation studies including the vector only (pMP814) or +*wbtJ* (pKM43; thatched bars) under these conditions. Significance was assessed using linear mixed effects model to compare the LVS wild-type to Isolate #15 and Δ*wbtJ* or strains containing pMP814 (vector only) to the isogenic strain harboring pKM43 (+*wbtJ* complement); ****P <0.0001. (b) Western blot analysis was performed to assess the LPS profile. Samples were suspended to an equal OD_600_, lysed, and separated on SDS-PAGE gels. After transferring to a membrane, blots were exposed to α-LPS (mAb FB11; top) and α-GroEL (bottom) was used as a loading control.

**Fig. 6.**
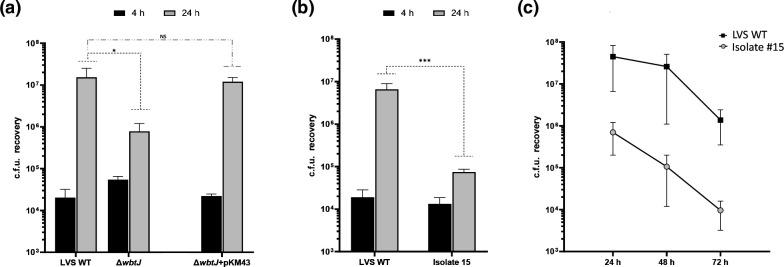
A Δ*wbtJ* mutant has a reduced fitness in J774A.1 macrophages and is rescued by complementation. Gentamicin protection assays were performed using the (a) LVS Δ*wbtJ* mutant and the complemented strain containing pKM43 and (b) the natural biofilm variant isolate #15. c.f.u. were determined at 4 h (black bars) and 24 h (grey bars) post infection. Error bars represent the standard error of the mean from three independent experiments. Statistical significance was determined using linear mixed effects model on log10 transformed data to compare each strain to the wild-type at each timepoint. *p<0.05; ***p<0.001. (c) J774A.1 macrophages were infected with isolate #15 and assayed over the course of 3 days to determine if replication was delayed in the biofilm forming variants.

**Fig. 7.**
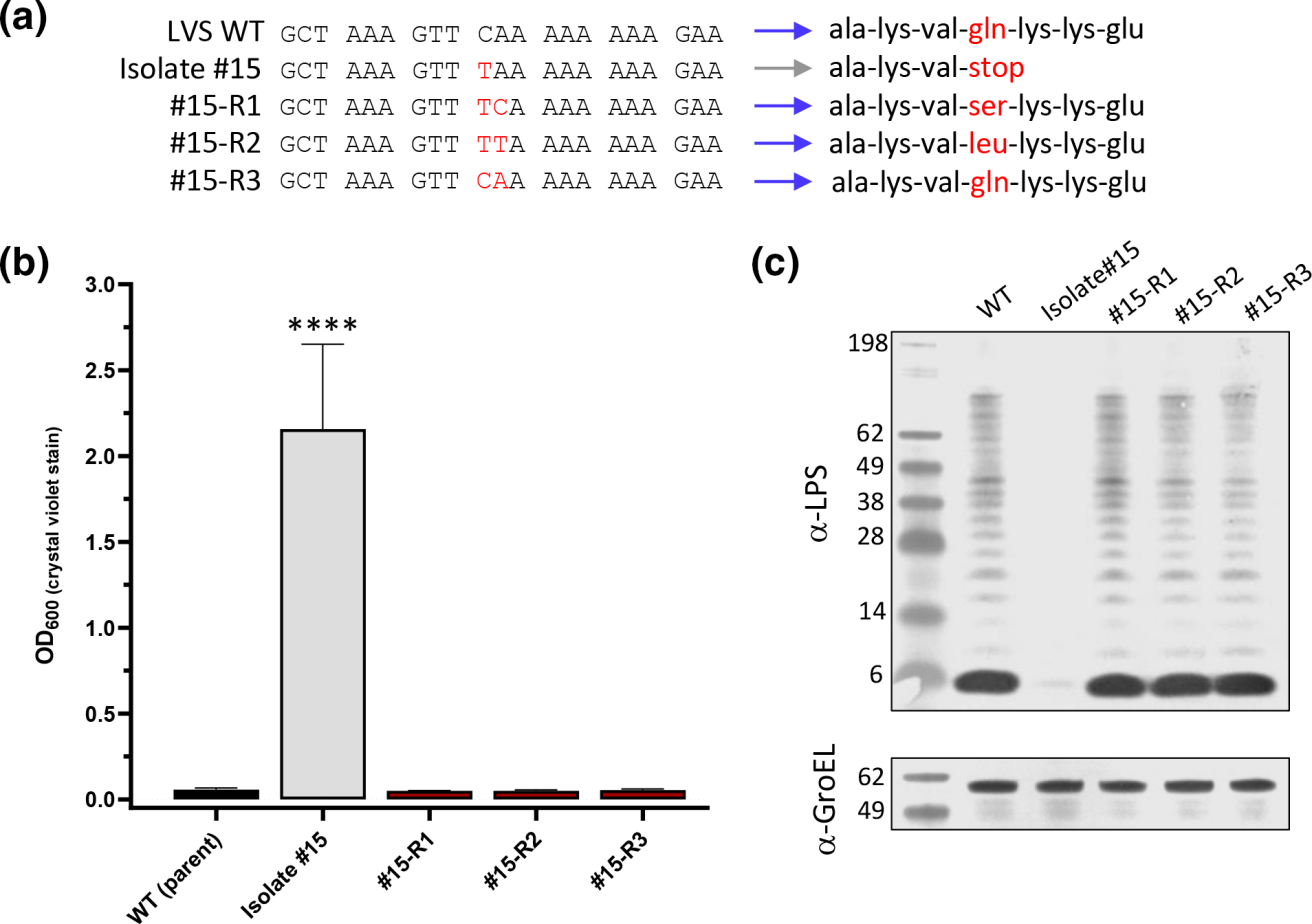
Natural reversion events within *wbtJ* can be detected in macrophage passaged isolate #15 samples. Heterogenous mixture of big and small colonies was observed in samples plated from macrophage assays with isolate #15 each time an experiment was conducted. Colonies that appeared larger than the parental isolate #15 were streak purified. (a) Sanger sequencing was performed of the *wbtJ* gene to determine the allele of the reverted isolate. Red font indicates a substituted nucleotide and the resulting codon compared to the LVS wild-type sequence for this gene. Blue and grey arrows indicate colony phenotype (b) The ability of the reverted isolates to form biofilm was assessed using crystal violet staining. For statistical analysis, a linear mixed effects model was used to compare each strain. ****p<0.0001. (c) The O-Ag was also assessed by western blotting performed with α-LPS (mAb FB11; top). α-GroEL (bottom) was included as a loading control.

**Fig. 8.**
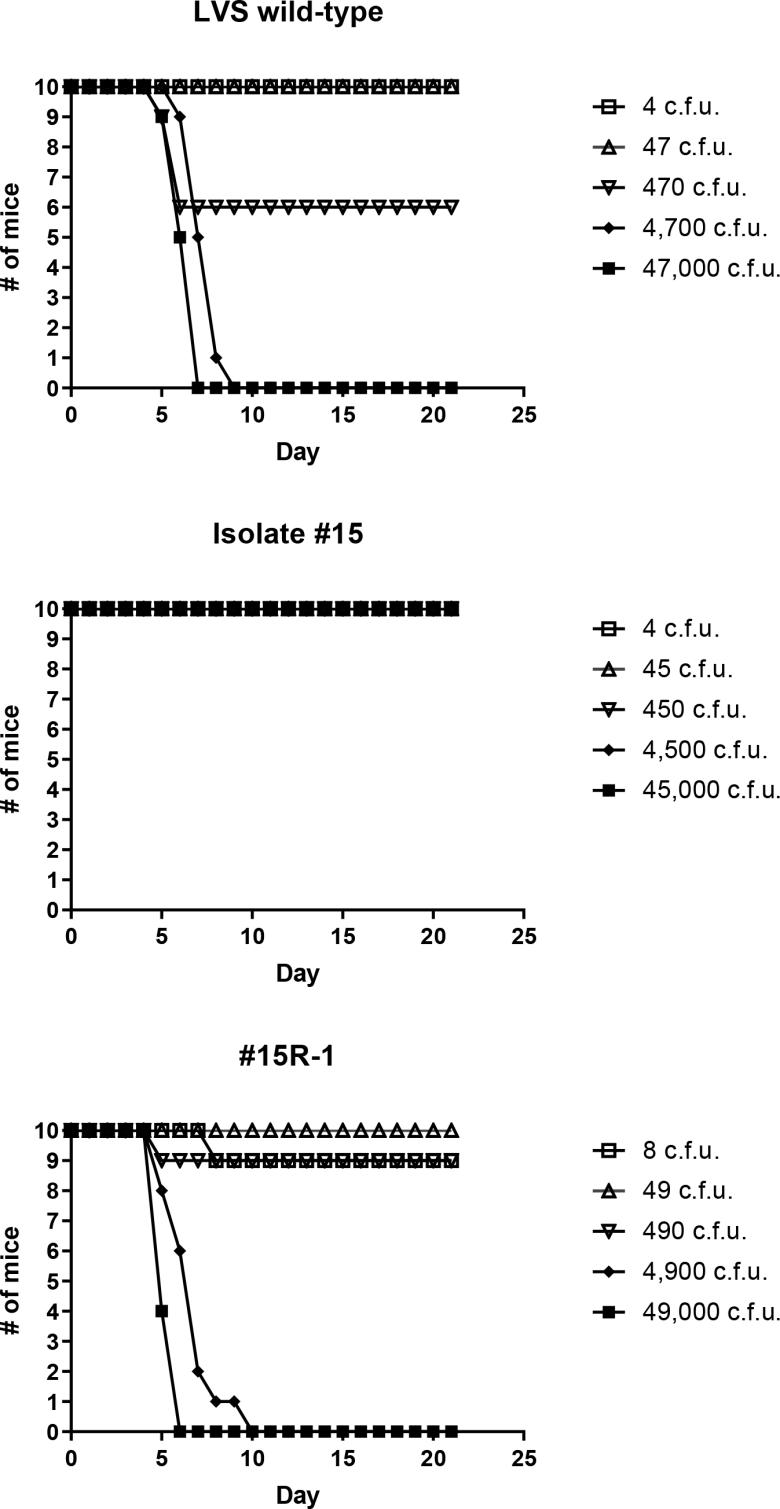
A natural biofilm-forming variant is completely attenuated in a pneumonic tularaemia mousemodel, but a reverted isolate displays wild-type virulence. Mice were challenged intranasally with doses ranging from 1 to 10^4^ c.f.u for the LVS wild-type (parent), isolate #15 and #15R-1. Displayed are the survival curves for groups of 10 mice following the mice for 21 days post challenge. The accompanying LD_50_ values and the median time-to-death calculated from these data are displayed in Table 5.

### Biofilm-forming variants display attenuation via subcutaneous and intradermal challenge in mice

The most common route of exposure to *F. tularensis* occurs through a bite from an infected arthropod [2]. Given that biofilm formation has been shown to be an important mechanism of persistence and transmission for other bacterial pathogens [41], we sought to first access the virulence potential of natural biofilm-forming *F. tularensis* variants. LD_50_ analysis was performed using mice challenged either subcutaneously (type A isolates) or intradermally (type B isolates) to simulate inoculation via either a tick or mosquito, respectively (Fig. 2) [22, 42]. Using this approach, the LD_50_ for both parental isolates (FRAN244 and FRAN255) was determined to be approximately 1–2 c.f.u. In stark contrast, mice exposed to the biofilm-forming variants for both FRAN244 and FRAN255 survived challenge at all doses (LD_50_ values >1000 c.f.u.). After 14 days, spleens were harvested from three mice from the highest challenge dose group (~10^3^) of each variant at random and plated to confirm bacterial clearance. All animals were sterile with the exception of one mouse infected with isolate 255 BF-4, from which 247 c.f.u./spleen were recovered.

### Biofilm-forming variants are highly attenuated in a pneumonic murine model

The most severe manifestation of tularaemia is the pneumonic form, which typically occurs following inhalation exposure [2]. With this in mind, we next assessed the virulence of the biofilm-forming variants by intranasally challenging mice and following survival for 14 days. The LD_50_ for BALB/c mice inoculated intranasally with wild-type *F. tularensis* strains is 1 c.f.u. [40], so a single challenge dose for the parental strains (FRAN244 : 105 c.f.u.; FRAN255 : 8 c.f.u.) was used as a comparison in this study (Fig. 3; red lines). For intranasal challenges, we calculated an LD_50_ value of 317 c.f.u. for 244 BF-1 and 2152 c.f.u. for 255 BF-4. An LD_50_ could not be determined for 255 BF-1, as we did not lose at least 50 % of the mice at any challenge dose. Collectively, all LD_50_ values for these biofilm-forming variants were still much higher than an LD_50_ of 1 c.f.u. for the respective parent strain. In addition, when comparing TTDs for those challenge doses that lead to the mice succumbing, the biofilm-forming variants had a significantly higher TTD when compared to the respective control strain (Table 2).

To determine whether bacteria could be recovered from the organs of mice that survived challenge with the variant strains, spleens and lungs were harvested and plated from five survivors challenged with ~10^2^ c.f.u. of 255 BF-1 (128 c.f.u.) or 255 BF-4 (680 c.f.u.). Bacterial c.f.u. were detected in both spleen and lung tissue for all animals assayed (approximately 500 and 1000 c.f.u. per organ, respectively). Notably, both large and small colony morphologies were observed on chocolate agar plates. However, further exploration of these noted select agent variants were not pursued at that time.

### Genetic analysis reveals that all biofilm-forming variants contain a mutation in *wbtJ*, a formyltransferase involved in O-antigen synthesis

As shown above, the *F. tularensis* variants 244 BF-1, 255 BF-1 and 255 BF-4 were highly attenuated in both *in vitro* and *in vivo* assays. In an effort to identify a possible genetic root cause for the observed grey variant phenotype, biofilm formation and the *in vivo* attenuation, we performed whole-genome sequencing on these *F. tularensis* strains. Strains were *de novo* assembled and aligned to their parental strain. When compared to the respective parental strain, the genomes of these strains lacked deleterious rearrangements or large-scale (>1 kb) deletions/duplication events (Fig. S2). Two identical inversions flanked by repetitive elements were found in 255 BF-1 and 255 BF-4, but coding sequences were not affected. To us, this suggested that a more subtle mutation may be responsible for the phenotypes associated with these strains. SNP analysis was performed, which revealed that the biofilm-forming variants were highly syntenic to the parental strain, harbouring fewer than 10 mutations across the genome (Table 3). Interestingly, each variant harboured a mutation within *wbtJ*, a gene encoding a formyl transferase located within an operon responsible for O-antigen synthesis [43]. A missense variant of *wbtJ* was identified in 244 BF-1 (Ser145Phe), while frameshifts were identified in *wbtJ* for 255 BF1/4 variants. Combined, these data strongly suggest that *wbtJ* is involved in both phase variation and biofilm formation and is important for virulence in *F. tularensis*.

To bolster the sample size for biofilm and variant genomic data, we again performed whole-genome sequencing followed by SNP analysis on five additional biofilm variants isolated independently in the LVS background that were previously described (LVS isolates #15, 22, 26, 27 and 38) [28]. In agreement with the findings for biofilm-forming variants of FRAN244 and FRAN255, each additional biofilm-forming variant of the LVS isolates also harboured a unique mutation within *wbtJ* (Fig. 4, Table 4). As a control, we also sequenced the genomes of three grey variants isolated from the same LVS heterogenous populations (LVS #11, 13 and 14) and performed SNP analysis. We previously demonstrated that these isolates do not form biofilm within 72 h and lack reactivity to αLPS specific to *F. tularensis* [28]. Once again, these non-biofilm-forming strains harboured very few mutations compared to the parental LVS strain (<5). In contrast to the biofilm-forming variants, these strains lacked aberrations within *wbtJ*, though SNPs were identified in genes known to be involved in LPS synthesis (Table S3). Based upon these genetic observations, we hypothesized that a nonfunctional WbtJ leads to both an O-Ag variant and a biofilm-forming phenotype. Additionally, other SNPs identified caused variant alleles of *wzy* (O-Ag polymerase) and *waaL* (O-Ag ligase) and *wbtA* (dTDP-glucose 4,6 dehydratase). Importantly, these genes are all involved in the synthesis of the O-Ag saccharides, but the mutations do not result in a biofilm-forming phenotype, which would suggest that general O-Ag dysfunction does not result in biofilm formation.

### A Δ*wbtJ* mutant recapitulates the phenotypes observed in natural biofilm forming variants

The *wbtJ* gene is a 726 bp ORF located in a locus encoding 15 genes involved in O-Ag synthesis that is flanked by insertion elements (Fig. 4). Disruption of genes (*wbtA*, *wbtC*, *wbtI* and *wbtM*) in this area via transposon mutagenesis has been reported by others to result in the loss of O-Ag on the LPS and capsule [44]. To determine whether the biofilm phenotype we observed in these naturally occurring variants was due solely to the inactivation of *wbtJ,* we constructed an in-frame deletion mutant (Δ*wbtJ*) in the wild-type background of LVS by allelic exchange. The final mutant was screened by PCR and Sanger sequencing to confirm the deletion of the *wbtJ* gene and ensure that no errors were generated to the upstream/downstream regions (data not shown).

When grown on a chocolate agar plate, the Δ*wbtJ* mutant recapitulated the colony morphology observed in the biofilm-forming variants and appears small and opaque as compared to the wild-type parent (data not shown). Additionally, the Δ*wbtJ* mutant appears to grow similarly to the parental LVS wild-type in CDM medium (Fig. S3). Given that we identified natural variants containing SNPs in *wbtJ* by their ability to quickly form biofilm, we first assayed the biofilm-forming capacity of this deletion mutant compared to the parental strain. We found that the Δ*wbtJ* mutant displayed the biofilm phenotype observed in a natural biofilm variant (LVS isolate #15), suggesting that lesions within the coding sequence of *wbtJ* in fact result in biofilm formation (Fig. 5a; *P*<0.0001; linear mixed effects model to compare Δ*wbtJ* to LVS wild-type). To demonstrate that the phenotype of the mutant was due specifically to the disruption of *wbtJ*, the complete coding sequence of *wbtJ* was cloned into shuttle vector pMP814 [39], and the resulting plasmid (pKM43) was electroporated into LVS *ΔwbtJ* and LVS isolate #15. In the *trans*-complemented strains, biofilm formation was alleviated in the transformed Δ*wbtJ* and LVS isolate #15 strains (Fig. 5a; *P*<0.0001; linear mixed effects model), consistent with mutations within *wbtJ* leading to the biofilm phenotype observed in natural variants and the *wbtJ* deletion strain.

We previously showed that the spontaneous variants used in this study lacked reactivity to a mAb generated against the O-Ag of *F. tularensis* [28]. Given that WbtJ is involved in the synthesis of the dTDP-4,6-dideoxy-4-romamido-d-glucose (Qui4NFm) saccharide that is incorporated into *F. tularensis* O-Ag [45], we hypothesized that the LVS Δ*wbtJ* mutant would also display this phenotype. Western blot analysis was performed using a mAb to *F. tularensis* LPS to assay whole-cell extracts obtained from the parent, Δ*wbtJ* mutant and complemented strains. As show in Fig. 5b, the results demonstrated that the null mutant did indeed lack reactivity for antibody specific to *F. tularensis* O-Ag and that complementation with an intact *wbtJ* gene restored the hallmark laddering pattern consistent with LPS (Fig. 5b).

### A Δ*wbtJ* mutant displays reduced fitness in macrophages

As shown above in Fig. 1, our natural biofilm-forming variants displayed an intracellular fitness defect at 24 h when compared to the parent strain. To determine whether this loss was due to the altered *wbtJ* gene in the variants, the Δ*wbtJ* mutant was tested for its ability to replicate within J774A.1 cells. Consistent with the natural variants, no differences were noted in c.f.u. recovery between the parent and deletion mutant strain at 4 h post-infection. In contrast, at the 24 h time point, the Δ*wbtJ* mutant did not reach a c.f.u. recovery comparable to the parent (Fig. 6a, grey bars; *P*<0.05, linear mixed effects model). Further, a complemented Δ*wbtJ* strain harbouring pKM43 was able to restore the c.f.u. recovered to wild-type levels, which was not observed in the vector-only control (pMP814; data not shown). Taken together, these results suggest that a functional copy of WbtJ is required for optimal fitness within host cells.

For completeness, we also tested the ability of a natural biofilm-forming variant in the LVS background to replicate within macrophages. Consistent with the results observed in the Δ*wbtJ* mutant, the c.f.u. recovery of isolate #15 was not as robust as that for the parental wild-type at 24 h post-infection (Fig. 6b, grey bars; *P*<0.001, linear mixed effects model). To ensure that the observed fitness defect was not due to delayed replication within macrophages, we assayed isolate #15 over the course of 72 h. In agreement with other studies, wild-type c.f.u. recovery was similar between 24 and 48 h post-infection [46], with a mild decrease observed at the 72 h sampling (Fig. 6c). An increase in c.f.u. recovery was not observed for isolate #15 at 48 and 72 h, which suggests that the survival defect identified in the biofilm-forming variants likely is not due to a delay.

### Natural biofilm-forming grey variants can revert to a ‘blue’ form due to repair of the *wbtJ* gene

While performing experiments using LVS-infected macrophages, we observed a low-level variation in the colony morphology in the natural biofilm-forming variants (<20 colonies in total per experiment, with an average of 1150 colonies counted), particularly at 24 h post-infection and beyond. Well isolated colonies were selected and streak-purified. Upon restreaking, the colony morphology appeared to resemble that of the LVS wild-type, as the colonies were larger and less opaque than those for LVS isolate #15 (data not shown). From separate experiments, three of these isolates (#15-R1, #15-R2 and #15-R3) were streak-purified and saved for further examination. Since reversion has previously been reported [18, 21], and it has been hypothesized to be the result of a secondary mutation rather than repair of the initial mutation (S→NS→S’ [21]), we performed Sanger sequencing on *wbtJ* for these three revertant strains to determine whether the original mutation (457C>T) was maintained. This examination revealed that not only was the original mutation present in #15 R-1 and #15 R-2, but a secondary SNP occurred directly next to this mutation that would eliminate the nonsense mutation introduced by the primary mutation (Fig. 7a). Isolate #15-R3 appeared to have the original mutation repaired to the wild-type sequence (Fig. 7a). Given that LVS isolate #15 formed a robust biofilm within 1 to 2 days, assays were performed on the reverted strains to determine whether biofilm formation would occur with these *wbtJ* alleles (Fig. 7b). Indeed, these strains had lost the ability to form biofilm and mirrored the LVS wild-type (*P*<0.0001; linear mixed effects model) Additionally, western analysis revealed that the parent O-Ag profile was restored in these isolates (Fig. 7c). Taken together, these results further suggest that the fitness of biofilm-forming variants within macrophages is impaired, and likely a selection pressure exists to favour a reversion event.

### Natural variants containing SNPs in *wbtJ* are highly attenuated via intranasal exposure but can revert to wild-type *in vivo*

Given that the natural LVS variant displayed a fitness defect in macrophages (though some level of replication still occurred), we sought to determine whether this natural variant (LVS isolate #15) and the reverted strain (#15 R-1) were virulent in mice via intranasal challenge. This analysis established that wild-type LVS has an LD_50_ of 509 c.f.u. with a median TTD of 6.5–7.5 days post-infection. Mice were challenged with LVS isolate #15 with doses ranging up to 45 000 c.f.u., and none displayed any signs of illness. Since all mice survived the challenge, we could not determine an LD_50_. However, virulence was restored to near wild-type levels in the reverted mutant #15 R-1 (LD_50_=815 c.f.u.), (Fig. 8) suggesting that virulence can be restored via phase variation.

At 21 days post-challenge, spleens were harvested from survivors receiving a dose of 10^2^ (~1 LD_50_ of wild-type) from both LVS wild-type and LVS isolate #15 to determine whether c.f.u. could be recovered. For the survivors challenged with the parental LVS strain (470 c.f.u.) remaining at day 21, these mice displayed no clinical signs; however, LVS (~200 c.f.u.) was recovered from all spleens. In contrast, spleens from animals challenged with LVS isolate #15 at a similar dose (450 c.f.u.) were found to be sterile. Additionally, spleens were harvested and plated from survivors challenged with 45 000 c.f.u. of LVS isolate #15. All animals within this group harboured an average of 174 c.f.u./spleen and featured a similar colony morphology to LVS wild-type. At random, 30 colonies encompassing all 10 spleens were restreaked and sequenced to analyse the *wbtJ* gene. As observed for macrophage studies, all restreaked isolates contained a ‘repaired’ allele of *wbtJ* with reversion to glutamine or leucine at residue 153 detected in these samples. Once again, biofilm formation was ablated and normal O-Ag banding was observed when western blot analysis was performed with these reverted isolates (Fig. S4). These results provide further evidence that reversion of the variants occurs *in vivo*.

### A Δ*wbtJ* mutant in LVS is attenuated in a murine pneumonic model of tularaemia

**Fig. 9.**
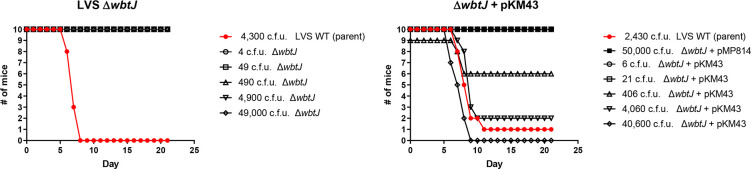
A Δ*wbtJ* mutant is completely attenuated in a pneumonic tularaemia mouse model and virulence can be restored by complementation. Groups consisting of 10 mice were challenged intranasally with a range of doses for either a Δ*wbtJ* mutant (left) or the complemented strain (right). As a control for these experiments, one group of mice was challenged with the LVS parent (red line). The mice were followed for 21 days post challenge. The accompanying LD_50_ values and the median time-to-death calculated from these data are displayed in Table 5.

We next assessed the virulence of the LVS Δ*wbtJ* strain and the corresponding complement Δ*wbtJ*+pKM43 using intranasal challenge in a mouse model. As a control for these experiments, mice were challenged with ~5 LD_50_ (2430 c.f.u.) of the parental LVS wild-type and a vector-only control (Δ*wbtJ*+pMP814) at a high dose (50 000 c.f.u.). In this experiment, we attempted to determine the intranasal LD_50_ for the Δ*wbtJ* mutant strain. However, mice challenged with a Δ*wbtJ* mutant displayed no clinical signs of illness even at the highest challenge dose (49 000 c.f.u.). In contrast, mice challenged with the complemented strain had an LD_50_ value of 933 c.f.u., demonstrating that WbtJ is required for virulence *in vivo* (Fig. 9, Table 5). At 21 days post-challenge, spleens from all mice challenged with Δ*wbtJ* were assayed for bacterial burden. For mice challenged with 490 c.f.u., only 1 animal had a detectable number of bacteria in the spleen (20 c.f.u.), while only 2 of the 10 mice from the highest challenge dose (49 000 c.f.u.) contained bacteria in the spleen (average of 25 c.f.u. per spleen). Each colony recovered from these samples were streak-purified, and the deletion of Δ*wbtJ* was confirmed via PCR. Notably, all mouse-passaged Δ*wbtJ* isolates recovered from this experiment maintained the ability to form biofilm and were grey varied by western analysis (Fig. S4, Table 6). Taken together, these data suggest that deletion of *wbtJ* can lock *F. tularensis* strains into a grey attenuated form.

**Table 5.** LD_50_ and TTD analysis for mice challenged intranasally with LVS variants and a Δ*wbtJ* mutant

		**LVS wild-type**	**Isolate #15**	**#15-R1**	**Δ** ***wbtJ***	**Δ** ***wbtJ*** **+ pKM43**
	LD_50_ (c.f.u.)	509	>45 000	815	>49 000	933
						
Median TTD (Days)	1–10	>21	>21	>21	>21	>21
	10–100	>21	>21	>21	>21	>21
	100–1000	>21	>21	>21	>21	>21
	1,000–10 000	7.5	>21	7	>21	9
	>10 000	6.5	>21	5	>21	7.5
						
						
Pair-Wise Comparisons (Wald-test)	vs. LVS wild-type		*P*<0.01	ns	*P*<0.01	ns

*P* values for comparison among strains were determined by Wald Chi-square tests based on a Cox regression model.

NS, Not Significant (*P*>0.05); TTD, Time to Death.

**Table 6.** Phenotypic summary of isolates recovered from challenged mice at 21 days post-challenge

Strain	Challenge dose (c.f.u.)	# of mice w/ detectable LVS	Avg c.f.u. Recovered*	Colony morphology	LPS status†	Biofilm Formation
						
LVS wild-type	470	5/5	204	Normal	nd	nd
						
						
Isolate #15	450	0/5	0			
	45 000	10/10	174	Normal	Blue form	Negative
						
Δ*wbtJ*	490	1/5	20	Small	Grey form	Positive
	49 000	2/10	25	Small	Grey form	Positive
						

*Based on plating homogenate from the complete spleen.

†As determined by western blot analysis using mAb specific to *F. tularensis* O-Ag.

## Discussion

Biofilm formation is considered to be a virulence factor among many pathogens, but there is little information available on how biofilm and pathogenesis intersect in *F. tularensis*. The goal of this study was to characterize the virulence of *F. tularensis* variants known to produce a robust biofilm within 24 h. The major finding of this work is that each of the biofilm-producing variants for both type A and B isolates used in this study displayed high levels of attenuation via multiple exposure routes in a murine model of tularaemia. We built upon this finding as we identified that mutation of the *wbtJ* gene was responsible for biofilm production in the natural variants and showed that a Δ*wbtJ* mutant recapitulated the associated phenotypes of the spontaneous variants. Additionally, we provided evidence that grey to blue variation occurs *in vivo* and show that reversion from the grey to the blue form allows *F. tularensis* to switch from an avirulent to a virulent state.

Previous studies utilizing murine macrophages to study intracellular survival have shown that grey variants remain as infective as wild-type since similar c.f.u. are recovered post-infection (between 0–4 h) but can differ in fitness once within host cells (12–24 h). For instance, the LVSG variant studied by Soni *et al.* within macrophage host cells did not display a survival defect, while the variant studied by Hartley *et al.* showed a reduced ability to survive intracellularly [23, 25]. Each of these studies utilized J774A.1 macrophages, which is where we began assessing virulence in the natural biofilm-forming *F. tularensis* isolates. Within the biofilm variants we tested, variability for intracellular fitness was also observed as 244 BF-1 and 255 BF-1 showed little replication, while c.f.u. recovery for 255 BF-4 at 24 h post-infection was comparable to wild-type. An advantage of our study over previous studies is that the variant genomes have been sequenced, allowing for reasonable conclusions about affected gene(s) or disrupted enzyme(s) causing grey variation and associated attenuation to be made.

Placing these results into the context of the genomic analysis of our strains for the *wbtJ* gene, 255 BF-4 contained a mutation further downstream of *wbtJ* than these other strains, which could potentially allow some functionality of the enzyme. The location of the *wbtJ* mutations (Fig. 4a) could also explain the observed pneumonic LD_50_ differences between the two FRAN255 variants when comparing 255 BF-1 (LD_50_>460 000) to 255 BF-4 (LD_50_=2151) (Table 2). It is also worth noting that in a previous study, 255 BF-4 was identified as unique due to some residual low-molecular-weight LPS being present by western analysis [28]. The observed LD_50_ differences in virulence between the various described grey variants in this study could be linked to the state of the LPS. In the case of Soni *et al.,* the LVSG mutant possessed full-length O-Ag, while the rough grey variant characterized by Hartley *et al.* appeared to lack O-Ag. It is likely that not all grey variants behave similarly despite a shared qualitative phenotype, as different genetic mutations can possibly result in different molecular O-Ag composition. Further analysis of the strains used in our study is currently being undertaken to better characterize the alteration of the O-Ag structure in biofilm-forming variants and the Δ*wbtJ* mutant.

Assessing the virulence by vector mimic showed that the variant strains were attenuated by at least a magnitude of 10^3^ when compared to the parental strain. These data also suggest that the route of exposure impacts on the virulence in these strains, as 244 BF-1 and 255 BF-4 were completely attenuated with doses of 10^3^ by subcutaneous and intradermal inoculation, but mice challenged intranasally were susceptible at this dose (compare Figs 2 and 3). It was also notable that all but one spleen was found to be sterile for mice challenged by vector mimic, while all mice challenged intranasally harboured bacteria within the spleen. A previous mouse challenge study with the wild-type parental isolates found that FRAN244 and FRAN255 displayed an average TTD of 4 and 6 days, respectively, when delivered at a dose between 10^3^–10^4^ c.f.u. intranasally [40]. Comparing those results to those of the current study, the biofilm-forming variants 244 BF-1 and 255 BF-1 TTD required nearly twice as long (8 and 11 days) at a dose between 10^3^ and 10^4^, respectively. Given that a low level of *F. tularensis* was present in the spleen homogenates, in hindsight it would be interesting to know whether these biofilm variants were reverted to the blue form. While multiple colony morphologies were observed in these spleen samples, no further characterization was performed at that time. However, since mice are exquisitely sensitive to *F. tularensis*, as the LD_50_ is normally ~1 c.f.u., reversion would presumably be lethal, which could account for the delayed TTD. To address this limitation, LVS was utilized in additional intranasal challenge studies, as this strain is attenuated at lower doses in mice, which could provide a window to detect reversion events.

In agreement with others, we have found that grey variants have a reduced fitness in mammalian host cells and *in vivo* [18, 23, 25]. We have extended these findings to include the link to phase variation and biofilm-forming isolates. It is still unclear how blue/grey variation of *F. tularensis* or biofilm formation factors into pathogenesis or survival in the environment. The blue form is the most represented isolate and considered the prototypical ‘wild-type’. Blue to grey variation has been known to occur on a scale of 10^−3^ and is readily observed in both type A and B isolates in a laboratory setting [21]. Perhaps variation to the grey form is an adaptation only observed under laboratory conditions, but this still remains to be determined. However, since the outcome of variation to grey leads to both an attenuated form and change in antigenicity, it is important to understand this phenomenon from a potential medical countermeasure perspective.

While certainly not exhaustive, the complete genomes of four LVS grey variants (isolates #11, #13, #14 and #15) were sequenced and were found to contain seemingly random SNPs in genes required for LPS synthesis rather than genomic rearrangements. While it was previously been observed that mutations within O-Ag synthesis genes quickly arise at a low frequency [47], it is puzzling why the variation would be at a minimum 100× more frequent than what would be expected if spontaneous mutations (rate of 10^−5^ to 10^−8^ [48]) were the sole reason for this variation. Complete genome sequencing of the eight biofilm-forming variants in this study (strains in Tables 3 and 4) detected only SNPs at a low frequency (1–6 per genome), which is consistent with the genomic stability reported for *F. tularensis* genomes [49, 50] and the results of studies on phase variation [51].

One possibility is that *F. tularensis* exists as a mixed blue/grey population in the natural environment, and this population shifts in an environment-dependent manner. For instance, robust biofilm is required for transmission of *Yersinia pestis* from a flea vector but is dispensable for pathogenesis within a mammalian host [41, 52]. Interestingly, the LPS of *Y. pestis* is also modified while maintained within a flea [53]. For *F. tularensis*, it is currently unknown if biofilm formation or phase variation occurs in an arthropod vector or if biofilm aids in persistence. Perhaps a mixed population is the predominant phenotype within a vector, but only blue forms are isolated from mammalian tularaemia infections as the grey form have a reduced fitness. Phenotypic plasticity is observed in *Burkholderia pseudomallei,* as the bacterium is thought to alter surface determinants, including the O-Ag, to facilitate survival [54, 55]. Further, variation of LPS in *B. pseudomallei* has been linked to persistence in an aquatic environment, ability to produce biofilm and host immune defence [56, 57]. With this in mind, a case can be made that variation of the LPS allows these Gram-negative intracellular pathogens to adapt to environmental or host reservoirs.

The emerging model from these data presented in this study is that grey variants can infect host cells but are impaired for replication or potential dissemination. This impairment then allows grey variants to persist, while reversion can occur, potentially tipping the infection toward lethality. Noteworthy, macrophages infected with our biofilm variants did not appear to undergo apoptosis as one would expect in response to *F. tularensis* strains having alterations to the LPS and/or O-Ag [58–60]. These data presented in this study support the theory put forth by Cowley *et al.* that phase variation can modulate intracellular growth to control host infection dynamics [18, 19].

To understand why these grey variants were constitutively producing biofilm, we performed whole-genome sequencing and, surprisingly, all biofilm-forming variants contained a mutation within *wbtJ* (eight in total). WbtJ is a *N*-formyltransferase that catalyses dTDP-4, 6-dideoxy-amino-d-glucose (dTDP-Qui4N) to dTDP-4, 6-dideoxy-4-formido-d-glucose (dTDP-Qui4NFm), which is incorporated into O-Ag. Within the *wbt* locus, *wbtI*, *wbtJ*, *wbtK*, *wbtL* and *wbtM* are genes unique to type A and B isolates that play a role in O-Ag synthesis [43, 61, 62] (Fig. 4). Namely, WbtL is thought to make activated dTDP-glucose, while WbtM is a dTDP-d-glucose 4,6 dehydratase; amination occurs through WbtI, resulting in dTDP-Qui4N, WbtJ formylates this sugar to become dTDP-Qui4NFm and WbtK functions as the glycosyltransferase to aid in biosynthesis of the O-Ag unit [43, 45, 61, 62]. Notably, blue/grey phase variation has not been observed in the closely related subspecies *Francisella novicida,* but the O-Ag unit differs in *F. novicida* compared to the virulent type A and B isolates [63, 64], and *F. novicida* lacks these five additional *wbt* genes [49, 65, 66]. While it is tempting to speculate that blue/grey variation is due solely to these additional genes, we detected spontaneous mutations within *wbtA*, *wzy* and *waaL* (Table S1), which are also encoded in the *F. novicida* genome. A previous study by Kurtz *et al.* performed genomic analysis on two different phase variants with altered LPS in an effort to understand immunogenicity, as LVS-G was moderately protective, while LVS-R provided little protection in mice [51, 67]. The exact mutation responsible for grey variation in these strains is unclear, although it is likely that a non-sense mutation in *FTL_0937* (polysaccharide biosynthesis) in LVS-G and *FTL_0429* (a glutamine amidotransferase) in LVS-R results in the associated phenotypes. This would suggest that an additional factor likely governs phase variation and may exert selective pressure to inactivate genes required for O-Ag synthesis or a regulatory network for LPS and cell surface antigens.

To deconstruct phase variation from biofilm formation, it is still unclear why inactivation of *wbtJ* specifically results in the production of a robust biofilm, and further studies are required to understand this phenomenon. One hypothesis that can be made from this work is that general dysfunction or ablation of surface polysaccharides can alter cell surface properties, such as charge, to promote attachment and/or cell-to-cell adhesion. However, if this were true, most grey variants would produce a robust biofilm. The enzymes encoded by *wzy* (mutated in isolate 13) and *waaL* (mutated in isolate 14) play a role in the polymerization and ligation of the assembled O-Ag unit, while *wbtA* (mutated in isolate 11) and *wbtJ* (mutated in isolate 15) are enzymes required to synthesize saccharides incorporated into the O-Ag [68]. It was previously shown that grey variation and O-Ag can affect the production of outer membrane vesicles (OMVs) [25, 69], which can have implications for biofilm formation, as increased OMV secretion has been linked to biofilm production [70]. LVS isolates #11, #13 and #14 do not form a biofilm, while a distinct extracellular matrix is present in biofilms produced by LVS isolate #15 [28]. The lack of biofilm formation by LVS isolate #11 (*wbtA* mutant) and LVS isolate #13 (*wzy* mutant) suggests that lack of a complete O-Ag or diminution of O-Ag repeat length does not account for the robust biofilm formation observed in *wbtJ* mutants. *F. tularensis* produces both O-antigen LPS and O-antigen capsule, both of which consists of repeating units of Qui4NFm-GalNAcAN-GalNAcAN-GuiNac. Mutations in *wbtA*, *wzy* and *waaL* are all thought to lack this structure [71]. While we have previously shown that stochastic biofilm formation through this specific phase variation mechanism lacks reactivity to mAb against capsule [28], it is unclear whether the capsule or capsule-like complex is present in *wbtJ* mutants and will require additional exploration in future studies.

We also found that a strong selective pressure exists to select for reversion events when multiple colony morphologies were identified in macrophage lysates and, more importantly, *in vivo* when determining the bacterial burden of spleen homogenates. While the complete genome of the reverted isolates was not sequenced, our data are the first to confirm the original hypothesis of Eigelsbach that restoration happens in the form of S → NS → S′ rather than S⇄ NS (smooth, non-smooth [21]). Simply stated, reversion occurs as a result of a secondary mutation masking the previous phenotype. A caveat to our study is that we relied upon LVS isolate #15, which harbours a SNP, to monitor reversion. We consistently identified three forms of the repaired *wbtJ* allele (encoding a ser, leu or gln) for reverted strains of LVS isolate #15 and wondered whether saturation was reached for the repaired alleles. However, other potential mutations that could arise at this codon would result in tyrosine (aromatic), lysine or glutamine (charged), or another stop codon. With this in mind, we suspect that the reverted isolates of LVS isolate #15 we identified are the most likely alleles that would repair WbtJ enzymatic function, especially considering codon 153 is in close proximity to the region that interacts with substrate [45].

It is likely that more deleterious mutations, such as deletion of multiple nucleotides, may be more difficult to repair and likely explain why some variants appear more stable than others [21, 33]. Thus, the in-frame deletion of Δ*wbtJ* LVS appears to have created a ‘locked’ grey variant that has not been observed to revert to the blue form. In addition, the Δ*wbtJ* mutant recapitulates the phenotypes associated with natural biofilm-forming variants. Construction of a locked variant is crucial to improving both the safety and the efficacy of live vaccine strains of *F. tularensis* [24]. Ideal candidates are thought to be variants locked in blue form [20, 67]; however, it is possible that a particular grey form may be able retain the immunogenic properties necessary for vaccination and display high levels of attenuation, as a systematic study of all the mutants responsible for phase variation has yet to be completed.

An additional consideration regarding biofilm formation in *F. tularensis* is that perhaps not all bacterial cells need to actively produce the extracellular matrix to reap the benefits offered by biofilm. Viewed in this light, stochastic variation of *F. tularensis* resulting in the ability to form biofilm is a novel example of division of labour in bacteria. It would be interesting, and important, to explore whether both blue and grey forms are encased within the extracellular matrix produced by biofilm-forming variants, especially considering that variation arises post-exponentially in ageing cultures [23, 28]. Given the stochastic nature of mutations causing variance, natural populations of *F. tularensis* may also be heterogenous to cope with changing environments, with each form differentially fitted for survival, resulting in an increase in overall fitness of the population. For instance, grey variants more readily form biofilm and stave off entering a viable but non-culturable state, which could enable better environmental persistence [28]. Grey variants, as well as a viable but non-culturable state, have been found to be attenuated in infection models, while blue variants are typically more fit within a host cell [72]. Bias towards blue fitness intracellularly has been demonstrated by using co-infection of macrophages with both blue and grey forms (1 : 10 ratio), with this resulting in the recovery of blue to grey at 100 : 1 [23]. These data presented in this current study further highlight this fitness difference as we detected reversion of grey variants to blue forms during infection.

In conclusion, the *wbtJ* gene has not previously been characterized in *F. tularensis*. We show that deletion of this gene causes *F. tularensis* to form robust biofilms and decreases the intracellular fitness of the bacterium. We further show that *wbtJ* is essential for pathogenesis in *F. tularensis.* The WbtJ enzyme could potentially be explored as a target for new antibiotics. Additionally, we have shown a link between the *wbtJ* gene and blue/grey variation that could potentially be exploited for medical countermeasure development to lock live vaccines into a particular variant form to aid in vaccine efficacy.

## Supplementary Data

Supplementary material 1
