# Supplementary material for: Mutation of wbtJ, a N-formyltransferase involved in O-antigen synthesis, results in biofilm formation, phase variation and attenuation in Francisella tularensis
Source: Microbiology (Reading). 2024 Feb 29;170(2):001437. doi: 10.1099/mic.0.001437 (PMC10924466; doi:10.1099/mic.0.001437)
Supplement: Supplementary material 1 [file mic-170-1437-s002.pdf]

Mutation of *wbtJ*, a *N*-formyltransferase involved in O-antigen synthesis, results in biofilm formation, phase variation, and attenuation in *Francisella tularensis*.

Kevin D. Mlynek[1#], Ron G. Toothman[1], Elsie E. Martinez[1], Ju Qiu[2], Joshua B. Richardson[3], Joel A. Bozue[1#]

[1]Bacteriology Division, [2]Regulated Research Administration Division, [3]Center for Genome Sciences, U.S. Army Medical Research Institute of Infectious Diseases (USAMRIID), Frederick, MD, USA

[#]co-corresponding authors: kevin.d.mlynek.civ@health.mil, joel.a.bozue.civ@health.mil

SUPPLEMENTARY MATERIALS

Materials and Methods

Table S1: Primers used in this study.

Primer Name	Sequence	Purpose
oKM189	ccc*GGATCC* ATTAATGATGATAGTCTTG	*wbtJ* (FTL_0602) proximal F for deletion (bamHI)
oKM192	ggtt*GATATC* GTAGTGTCGTTTGTATGATAC	*wbtJ* (FTL_0602) distal R for deletion (ecoRV)
oRGT_wbtJ_SOE1R	cccgggcccgggcccCATGAGGAAACCTCTTTGATATTATTA	deletion of *wbtJ* upstream flank use with oKM189
oRGT_wbtJ_SOE2F	gggcccgggcccgggTAGAAAAATGAGCCTTAAAAAAAATAC	deletion of *wbtJ* downstream flank use with oKM192
oKM193	ccc*GGATCC* CGATAAAATTAATAAAATAATT	*wbtJ* (FTL_0602) Forward complementation (ecoRV)
oRGT_wbtJ_seq F	gagccaaagcagtatatgccaa	Sanger sequencing of *wbtJ* region
oRGT_wbtJ_seq R	caatcagaccaaatgcatcatgac	Sanger sequencing of *wbtJ* region

Table S2: Plasmids used in this study.

Plasmid Name	Description	Source
pEDL50	suicide vector for allelic exchange	Lovullo et al., 2012
pKM41	pEDL50 containing *wbtJ* deletion fragment	This study
pMP814	empty vector with *blaB* promoter	Lovullo et al., 2009
pKM43	pMP814 containing a functional copy of *wbtJ*	This study

Generation of growth curves. *F. tularensis* strains were resuspended in PBS (phosphate buffered saline, pH 7.2) to an OD_{600} of 0.3. Bacterial suspensions were diluted 1 to 10 into or Chamberlain's Defined Medium (CDM)[1] in a 96-well plate. Growth was then assayed by OD_{600} reading every 30 min using a Tecan M200 Pro (Tecan Systems, San Jose, CA) microplate reader at 37°C with orbital shaking. Absorbance values were determined using the average of triplicate wells and subtracting the medium background as determined by the sterility control.

<u>Results</u>

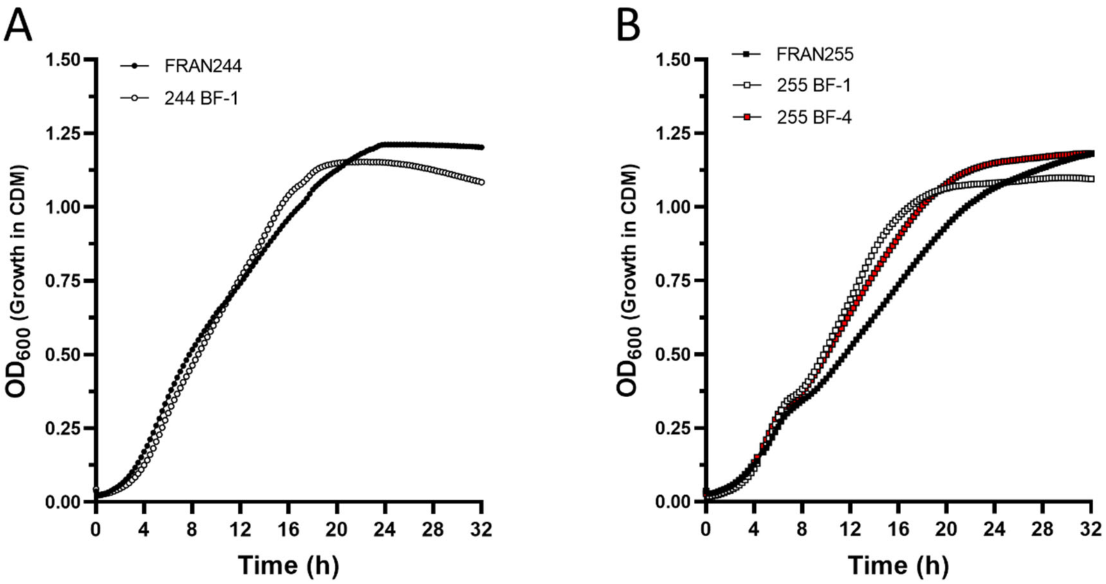

Figure S1: Growth curve analysis of biofilm forming variants compared to the parental isolate. (**A**) FRAN244 or (**B**) FRAN255 variant isolates were cultured in CDM at 37°C shaking over the course of 32 h using the OD_{600} to monitor growth. Data are the average of 2 independent experiments based upon 3 technical replicates per assay.

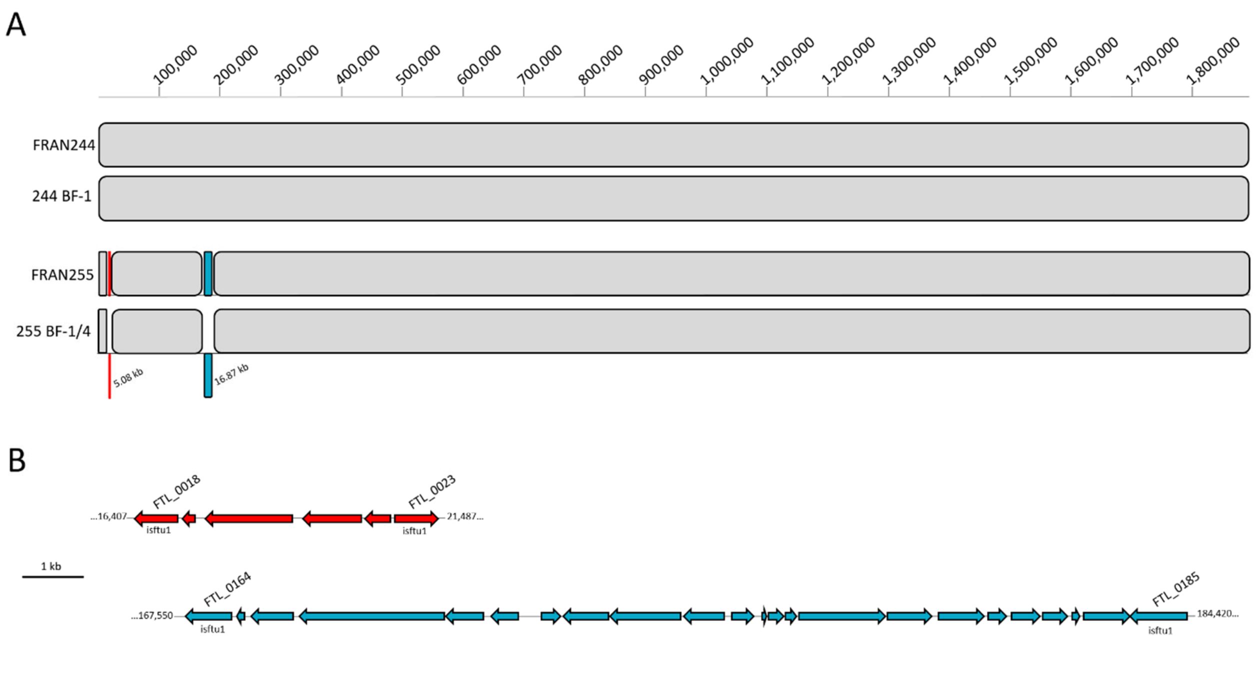

LVS genome co-ordinates displayed for ease of comparison   (NC_007880)

Figure S2: Genome alignment of biofilm forming variants. Illumina and Nanopore reads were used to generate *de novo* assemblies via Unicycler. Mauve was used for whole genome alignments compared to the respective parental strain. Two identical inversions were identified in 255 BF-1 and 255BF-4 bounded by repetitive mobile elements (isftu1) spanning 5080 bp (red invert) and 16, 870 bp (blue invert). For comparison purposes, LVS genome co-ordinates are shown.

Table S3: SNPs identified in LVS gray variants that do not form biofilm as a control comparison.

STRAIN	PHENOTYPE	POSITION	REF	ALT	STRAND	NT POS	AA POS	EFFECT	MUTATION	LOCUS TAG	KEGG	SchuS4 HOMOLOG	GENE	DESCRIPTION
LVS Isolate #15	GV, biofilm +	108388	C	T				intergenic		AW21_128	FTL_1339	FTT_0572		potential promotor alteration
		1315079	C	T	-	457/726	153/241	nonsense	Gln153*	AW21_1477	FTL_0602	FTT_1454c	*wbtJ*	formyl transferase
		1848663	G	A		14/115		unknown		AW21_2092	FTL_R0032		*rrf*	encodes 5sRNA
LVS Isolate #11	GV, biofilm -	1303079	G	T	-	292/1737	98/579	Nonsense	Glu98*	AW21_1467	FTL_0592	FTT_1464c	*wbtA1*	polysaccharide biosynthesis protein
LVS Isolate #13	GV, biofilm -	960093	T	G	+	458/624	153/208	missense	Val153Gly	AW21_1081	FTL_0237	FTT_0326	*rplD*	50s ribosomal protein L4
		1309532	T	G	-	194/1230	65/410	missense	Phe65Cys	AW21_1473	FTL_0598	FTT_1458c	*wzy*	Membrane protein/O-antigen protein
		1636790	C	A	+	423/633	141/211	synonymous	Ala	AW21_1838	FTL_0942	FTT_0668		nicotinamide mononucleotide transporter pnuC
LVS Isolate #14	GV, biofilm -	452704	G	T	-	348/474	116/158	synonymous	Gln	AW21_509	FTL_1693	FTT_1625c		putative pilus assembly protein
		1419600	T	C	+	449/1272	150/424	missense	Phe150Cys	AW21_1590	FTL_0705	FTT_1239		putative membrane protein
		1420302	C	T	-	47/1332	16/444	missense	Ala16Val	AW21_1591	FTL_0706	FTT_1238c	*waaL*	o-antigen ligase like membrane family protein

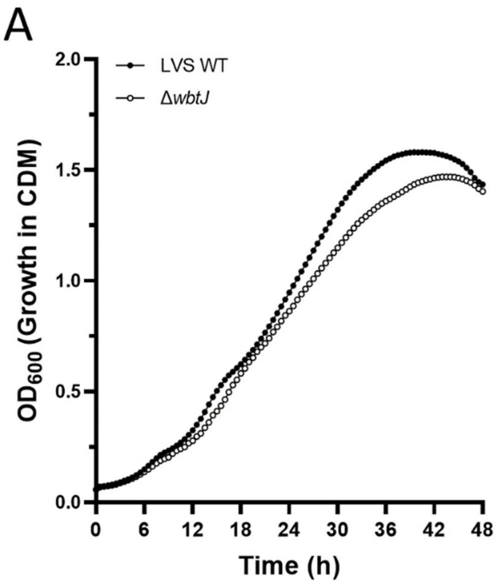

Figure S3: Growth curve analysis of an in-frame Δ*wbtJ* deletion mutant compared to the parental isolate. LVS wild-type (parent, black circles) or an isogenic Δ*wbtJ* mutant (open circles) was cultured in CDM at 37°C shaking over the course of 48 h using the OD_{600} to monitor growth. Data are the average of 3 independent experiments based upon at least 3 technical replicates per assay.

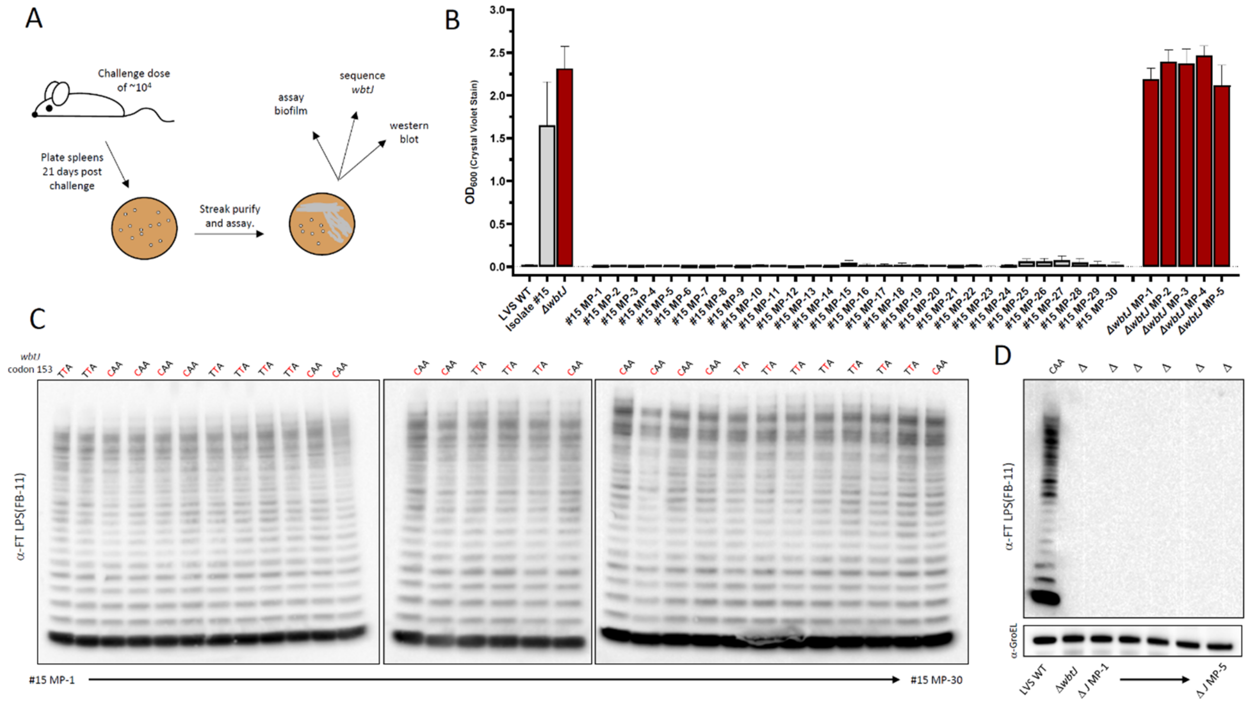

Figure S4: Phenotypic assessment of colonies recovered from mouse spleen at 21 days post challenge for Isolate #15 and a Δ*wbtJ* mutant. (**A**) A schematic illustrating how samples were recovered an analyzed at the end of study for Isolate #15 and a Δ*wbtJ* mutant to determine the wbtJ allele, biofilm forming capacity, and O-Ag status. (**B**) Biofilm formation of purified representative isolates recovered from mice spleens after challenge. Crystal violet staining was performed at two days post inoculation after static incubation at 37°C. Western analysis with α-LPS (FB-11) on mouse passaged samples from (**C**) isolate #15 and (**D**) Δ*wbtJ* mutant. The sequence at the codon mutated in *wbtJ* is indicated above each lane. In the case of Δ*wbtJ* isolates, α-GroEL was used as a loading control and *wbtJ* was confirmed to be absent by sequencing. These data support the summary data provided in Table 6.

<u>References</u>

1. Chamberlain, R.E. Evaluation of Live Tularemia Vaccine Prepared in a Chemically Defined Medium. *Appl Microbiol* 13, 232-235 (1965).
